# Falling Direction can Predict the Mechanism of Recurrent Falls in Advanced Parkinson’s Disease

**DOI:** 10.1038/s41598-017-04302-7

**Published:** 2017-06-20

**Authors:** Jinyoung Youn, Yasuyuki Okuma, Minho Hwang, Dongyeop Kim, Jin Whan Cho

**Affiliations:** 1 Department of Neurology, Samsung Medical Center, Sungkyunkwan University School of Medicine, Seoul, Republic of Korea; 2 Neuroscience Center, Samsung Medical Center, Seoul, Korea; 3 Department of Neurology, Juntendo University Shizuoka Hospital, Shizuoka, Japan

## Abstract

Falls are a common and disabling symptom in patients with Parkinson’s disease (PD). For prevention, it is important to understand the pathophysiology of falls in PD patients, but the predictors for the possible mechanisms underlying such falls have not been clearly elucidated. In this prospective observational study, we investigated the implications of falling direction to predict the mechanisms of recurrent falls in PD patients. We enrolled 62 recurrent fallers with PD and divided them into two groups according to the main falling directions: 45 PD fallers who fell forward (forward fallers), and 17 PD fallers who fell in the other directions (non-forward fallers). Although there was no difference in demographic data, parkinsonism, or frontal lobe function, forward fallers showed more severe falls and tended to fall during walking or turning, while non-forward fallers usually fell during sitting/standing or turning. Additionally, forward fallers revealed higher score on a freezing of gait (FOG) questionnaire. Logistic regression analysis demonstrated that FOG was associated with falling forward, while balance impairment, akinetic-rigid subtype, and neuropsychiatric symptoms were associated with falling into the other directions. Our results indicate that FOG and balance impairment are two major mechanisms for recurrent falling in PD patients, and falling direction is an important predictor for these mechanisms.

## Introduction

Falls are common symptom in Parkinson’s disease (PD), and present even from early disease stage^1–3^. Furthermore, falling could be disabling^4^, and disturb the quality of life in PD patients^5, 6^. To prevent falls in PD patients, it is crucial to understand the pathophysiology of falls, but the mechanisms have not been clearly elucidated.

Falling in PD patients happens in various situations^7^, and diverse parkinsonian motor and non-motor symptoms, including mood and cognitive disorders, have been reported to be associated with falls in PD patients^2, 8–12^. Additionally, PD patients fall in multiple directions, and different body parts are injured during such falls^5, 10^. Therefore, falls in PD patients should be regarded as a heterogeneous symptom with multiple mechanisms and it is important to divide falls into subtypes by possible mechanisms. However, despite many previous studies, no clinical markers for identifying subgroups of falls have been investigated^2^.

Among the aforementioned related factors, we hypothesized falling direction is a relevant predictor for the mechanism of recurrent falls in PD patients. Although the majority of PD patients tend to fall forward, previous studies have reported various falling directions and the relationship between falling direction and fracture site^5, 13^. However, no study has focused on the mechanism of falls based on the falling direction in PD patients. Moreover, unlike laboratory or imaging tests, falling direction can be easily accessed with a simple question.

In this study, to investigate the mechanisms of falls in PD patients, we divided recurrent PD fallers into subgroups based on their main falling direction and compared the clinical characteristics between these two subgroups.

## Methods

### Subjects

This study was approved by the Institutional Review Board of Samsung Medical Center, and all enrolled subjects provided written informed consent. All methods were carried out in accordance with the relevant guidelines and regulations. We enrolled recurrent PD fallers from December 2014 to June 2015 at the Movement Disorders Clinic at Samsung Medical Center, Seoul, Korea. All PD patients were diagnosed using the United Kingdom Parkinson’s Disease Society Brain Bank criteria^14^, and recurrent fallers were classified as PD patients who had fallen two or more times during the past 6 months^2^. We divided recurrent PD fallers into two groups based on their main falling direction: forward PD fallers (recurrent PD fallers who mainly fell forward) and non-forward PD fallers (recurrent PD fallers who mainly fell backward or sideways).

We excluded subjects with orthostatic hypotension (at least a 20 mmHg fall in systolic blood pressure and/or 10 mmHg fall in diastolic blood pressure within 3 minutes of head-up tilt test at 70° on a tilt table)^15^, cognitive impairment (Mini-Mental State Examination score < 20 or fulfillment of *DSM-IV* criteria for dementia), sedative medications associated with gait disturbance, or other medical conditions that could affect gait, including depression, stroke, ataxia, apraxia, atypical parkinsonism, or joint problems (knee, hip, or spine).

### Clinical evaluations

Demographic and clinical data were collected from all enrolled subjects. Parkinsonism was evaluated with the Unified Parkinson Disease Rating Scale (UPDRS) part 3 and the Hoehn and Yahr (HY) stage^16^. Subtypes of PD were defined as akinetic-rigid, tremor dominant and mixed subtypes^17^. We verified wearing off and dyskinesia with UPDRS part 4 and other PD-related symptoms, such as Pisa syndrome or camptocormia, with a movement disorders specialists. The daily levodopa equivalent dose (LED) was calculated for all enrolled subjects based on a previous study^18^. To investigate gait and balance problems, freezing of gait (FOG) was assessed using the FOG questionnaire^19^, and balance with the Tinneti balance assessment tool^20^. The Frontal Assessment Battery (FAB) was used to evaluate frontal lobe dysfunction^21^, and the Neuropsychiatric Inventory (NPI) was used to assess neuropsychiatric function^22^. Additionally, to identify characteristics of falls, we compared falling severity, frequency, and situations between forward and non-forward fallers. The severity was defined as mild (did not need any treatment), moderate (needed simple treatment) and severe (needed to visit the hospital). We used item A.1.12 of the Gait and Falls Questionnaire to evaluate falling frequency^19^, and evaluated common falling situations with three conditions: sitting/standing, walking and turning.

### Statistical analysis

All data were presented as mean and standard deviation. Demographic and clinical data were compared between forward and non-forward PD fallers using an independent t-test or Mann-Whitney U-test for continuous variables and Pearson’s χ^2^ or Fisher’s exact test for categorical variables. To investigate the characteristic features associated with falling direction, multivariate analysis was carried out using a logistic regression model with demographic and clinical data as independent variables and falling direction as the dependent variable. The results of the logistic regression were presented using 95% confidence intervals (CIs). To elucidate the correlated factors with falling frequency and severity in the two groups, partial Spearman correlation analyses were carried out between variables about falls (falling severity and frequency) and parkinsonism (parkinsonism, FOG severity and frequency, and balance impairment) with other clinical data as controlled variables, including age, gender, disease duration, LED, the presence of Pisa syndrome or camptocormia, dyskinesia, wearing off, FAB and NPI scores. *p*-values < 0.05 were considered statistically significant. Statistical analyses were performed with a commercially available software package (PASW version 18.0; SPSS Inc., Chicago, IL, USA).

## Results

We enrolled 62 recurrent fallers with PD in this study. All enrolled subjects were divided into two groups based on main falling direction; 45 (72.6%) were forward PD fallers, and 17 (27.4%) were non-forward fallers. Of all 17 non-forward fallers, 11 fell mainly backward, while six fell mainly sideways.

Demographic and clinical data between two groups are shown in Table 1. The only significant difference was seen in variables about FOG, not in the other variables including UPDRS part 3 score, posture problems, motor subtype, dyskinesia, wearing off, or frontal lobe dysfunction. Forward fallers showed significantly more severe FOG than non-forward fallers. Additionally, forward fallers also reported more frequent FOG compared with non-forward fallers, but this difference was not statistically significant.

**Table 1:** Demographic and clinical data of recurrent fallers with Parkinson’s disease.

	Total fallers (n = 62)	Forward fallers (n = 45)	Non-forward fallers (n = 17)	*p*-value
Age, years	70.5 ± 7.9	69.9 ± 7.9	72.2 ± 8.0	0.602
Gender, male/female	32/30	25/20	7/10	0.739
Disease duration, years	11.3 ± 4.1	11.4 ± 4.1	10.8 ± 4.4	0.632
LED, mg/day	876.0 ± 359.3	904.8 ± 386.5	800.0 ± 270.0	0.236
HY (On)	2.3 ± 0.7	2.3 ± 0.7	2.4 ± 0.7	0.595
UPDRS part 3	22.5 ± 9.3	23.0 ± 9.8	21.1 ± 8.1	0.447
Subtype, TD/AR/MX, n(%)	8/54/0 (12.9/87.1/0)	7/38/0 (15.6/84.4/0)	1/16/0 (5.9/94.1/0)	0.427
Camptocormia, n (%)	9 (14.5)	5 (11.1)	4 (23.5)	0.242
Pisa syndrome, n (%)	13 (21.0)	9 (20)	4 (23.5)	0.242
Dyskinesia, n (%)	21 (32.8)	16 (32.7)	5 (33.3)	0.551
Wearing off, n (%)	34 (54.8)	26 (57.8)	8 (47.1)	0.570
FOG, n (%)	46 (74.2)	36 (80)	10 (58.8)	0.111
FOGQ total score	11.2 ± 5.2	12.2 ± 5.0	8.7 ± 5.2	0.022
FOGQ, sum of items 3-6	6.5 ± 4.3	7.3 ± 4.2	4.4 ± 4.1	0.019
Tinnetti balance score	12.8 ± 3.3	12.9 ± 3.2	12.4 ± 3.4	0.514
FAB	12.4 ± 3.2	12.9 ± 2.9	12.2 ± 3.1	0.475
NPI	9.8 ± 13.6	8.1 ± 12.2	14.2 ± 16.2	0.106

PD, Parkinson’s disease; LED, daily levodopa equivalent dose; HY, Hoehn and Yahr stage; UPDRS, unified Parkinson’s disease rating scale; FOG, freezing of gait; FOGQ, freezing of gait questionnaire; FAB, frontal assessment battery; NPI, neuropsychiatric inventory.

All the characteristics of falls between the two groups are presented in Table 2. Forward fallers had more severe falls, while non-forward fallers showed milder falls. Particularly, 2/3 of non-forward fallers reported mild falls which did not need any treatment. In terms of falling frequency, falls happened more frequently in forward fallers than non-forward fallers, but this did not reach statistical significance. Based on these results, forward fallers could be considered to have more severe and frequent falls compared with non-forward fallers. Additionally, forward fallers and non-forward fallers reported different falling situations. Most of forward fallers reported falls during walking and turning, while non-forward fallers reported falls during standing/sitting and turning.

**Table 2:** Characteristics of falls between forward and non-forward fallers with Parkinson’s disease.

	Total fallers (n = 62)	Forward fallers (n = 45)	Non-forward fallers (n = 17)	*p*-value
Falling severity				0.019
Mild, n (%)	32 (51.6)	21 (46.7)	11 (64.7)	
Moderate, n (%)	22 (35.5)	18 (40)	4 (23.5)	
Severe, n (%)	8 (12.9)	6 (13.3)	2 (11.8)	
Falling frequency				0.052
Very rare, less than 1/month	21 (33.9)	16 (35.6)	5 (29.4)	
Rarely, 1/month ~ 1/week	15 (24.2)	10 (22.2)	5 (29.4)	
Often, 1/week ~ 1/day	8 (12.9)	3 (6.7)	5 (29.4)	
Very often, once a day or more	18 (29.0)	16 (35.6)	2 (11.8)	
Falling during				0.003
Sitting/Standing, n (%)	11 (17.7)	3 (6.7)	8 (47.1)	
Walking, n (%)	8 (12.9)	7 (15.6)	1 (5.9)	
Turning, n (%)	39 (62.9)	32 (71.1)	7 (41.2)	
Others, n (%)	4 (6.5)	3 (6.7)	1 (5.9)	

**Figure 1:**
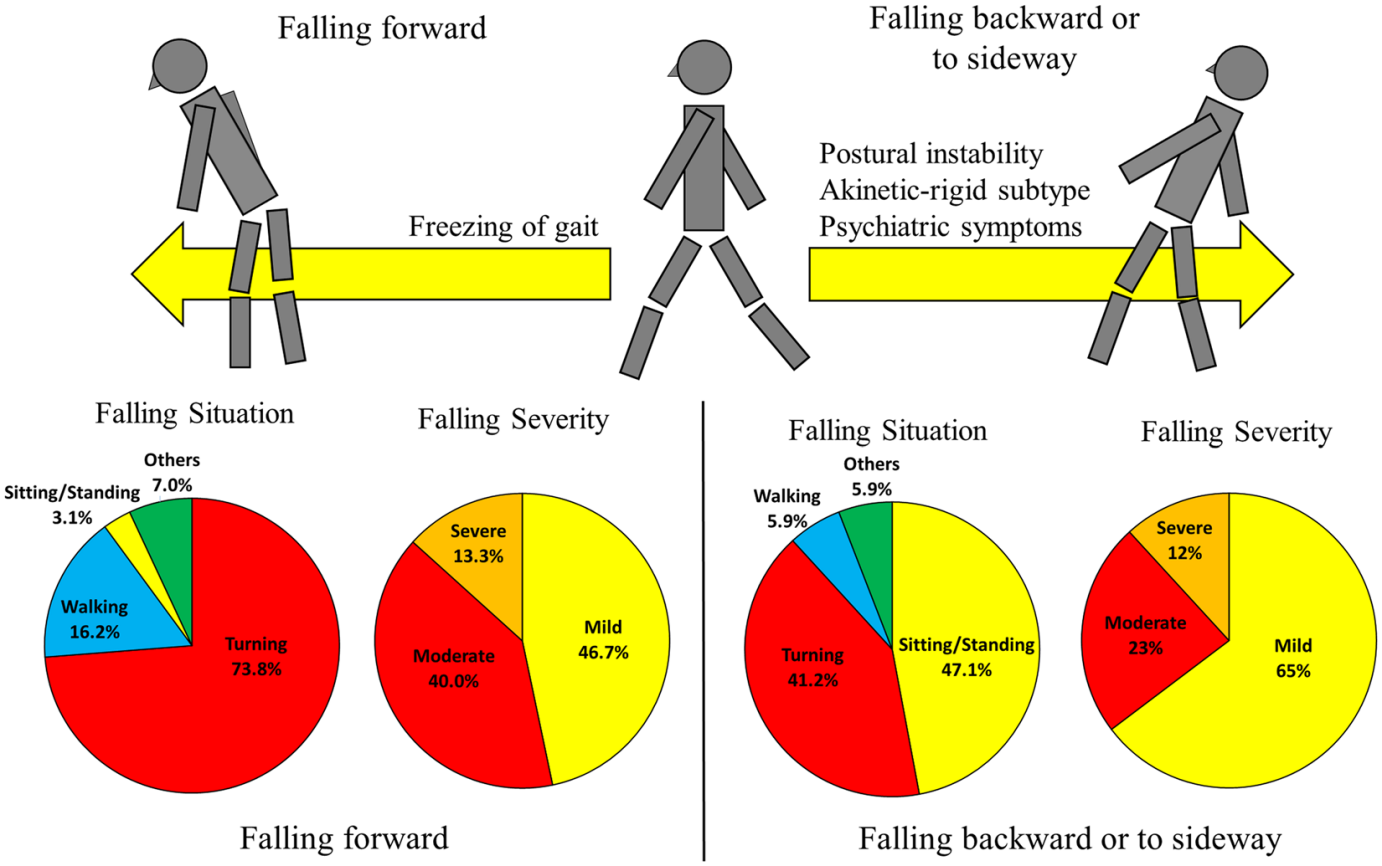
Scheme of the mechanism and characteristics of PD forward fallers and non-forward fallers. Falling forward was related to freezing of gait, while balance impairment, akinetic-rigid subtype, and neuropsychiatric symptoms were associated with falling backwards or sideways. In terms of falling situations, forward fallers tended to fall during walking and turning, while non-forward fallers mainly fell during walking and sitting/standing. Additionally, non-forward fallers had more frequent mild falls, and less moderate falls compared with forward fallers.

To identify the predictors associated with falling direction in recurrent PD fallers, we performed logistic regression analysis. Age, gender, disease duration, LED, UPDRS part 3 score, motor subtype, Pisa syndrome, camptocormia, wearing off, dyskinesia, Tinneti balance score, sum of FOGQ items 3-6, FAB, and NPI score were included as independent variables, and falling direction (forward and non-forward) as the dependent variable. Logistic regression analysis showed four variables were associated with falling direction. Higher sum score of FOGQ items 3-6 was a predictor for falling forward (OR = 1.469 (1.114-1.937 for 95% CIs), and p = 0.006), while non-forward falling was related with poor Tinneti balance score (OR = 1.417 (1.004-2.000 for 95% CIs), p = 0.047), higher NPI score (OR = 1.089 (1.013-1.172 for 95% CIs), p = 0.022) and akinetic-rigid subtype (OR = 115.979 (1.715-7843.500 for 95% CIs), p = 0.027). The related factors and characteristics with falling directions are illustrated in Fig. 1.

In each group, we performed correlation analysis to figure out the related factors with falling frequency and severity. In forward fallers, both frequency (FOGQ item 3 score) and severity (sum of FOGQ item 3-6) were significantly correlated with falling frequency, but not with falling severity (Table 3). In contrast, we did not find any factors significantly associated with falling severity or frequency in non-forward fallers.

**Table 3:** Correlation analysis between fall variables in forward fallers, and other variables including FOG, balance, and parkinsonism, controlling for age, gender, disease duration, LED, Pisa syndrome, camptocormia, dyskinesia, wearing off, FAB and NPI scores.

	Falling severity		Falling frequency	
	r	*p*-value	r	*p*-value
UPDRS part 3 score	−0.980	0.574	0.281	0.102
Tinneti balance scale score	0.005	0.997	−0.283	0.099
FOGQ item 3 score	0.197	0.256	0.349	0.040
Sum of FOGQ item 3-6	0.270	0.117	0.380	0.024

FOG, freezing of gait; LED, daily levodopa equivalent dose; FAB, frontal assessment battery; NPI, neuropsychiatric inventory; UPDRS, unified Parkinson’s disease rating scale; FOGQ, freezing of gait questionnaire.

## Discussion

This is the first study to suggest a relationship between falling direction and possible mechanisms of recurrent falls in PD patients. Considering that falling is an heterogeneous symptom in PD patients^23^, it is important to divide PD fallers into proper subgroups in order to understand fall mechanisms. Even though many previous studies have tried to identify factors related to falls in PD patients^2, 3, 8–10, 23^, only a few studies have focused on subgroups of fall types^2, 5^, and no study divided falls based on falling direction.

In this study, we identified two major mechanisms underlying recurrent falls in PD patients; FOG and balance impairment. Similarly, FOG and balance impairment were also suggested as major factors related to falls in previous studies^23–25^, but none of them investigated the relationship between the aforementioned mechanisms and falling direction.

We demonstrated FOG was predictor for falling forward. When FOG occurs in PD patients, their center of gravity continues to move forward when their feet stop moving, leading to falling forward if they cannot compensate with protective steps^26^. In addition, considering that PD patients with FOG usually fall forward, they have a fear of falling forward^26^, and tend to lean backward during standing in order to compensate for falling forward^27^. Therefore, to initiate walking, PD patients with FOG require wider posterior-to-anterior movements than those without FOG, and this wider movement in the forward direction can result in a forward fall. Similarly, anterior-posterior swaying was reported as one of the predictors of falls in PD patients^23^.

Interestingly, forward fallers showed more severe FOG than non-forward fallers, but there was no difference in the frequency of FOG between two groups. Furthermore, we showed that the frequency and severity of FOG was correlated with falling frequency in forward fallers, but not in non-forward fallers. Based on these results, we suggest that the severity of FOG is important, and more severe FOG can cause more forward falls in PD patients. In accord with these results, a previous study also demonstrated FOG score predicted falls in PD patients^23^. Therefore, even though PD patients with mild FOG may not fall, FOG should be carefully assessed and managed, because such patients could fall forward if their FOG worsens.

Previous studies have shown balance impairment is closely associated with falls and mobility in PD patients^28, 29^. Additionally, using various scales, balance problems could be easily checked in PD patients, and it is possible to predict falls with these scales^30^. Therefore, balance function is crucial to avoiding falls, and prevention strategies focusing on postural instability, such as balance rehabilitation, could reduce falls in PD patients^31, 32^. However, the relationship between falling direction and balance impairment in PD patients was not studied previously.

When we evaluated the situations for falling, non-forward fallers reported that falls occurred mainly during standing/sitting or turning, while forward fallers reported falls during walking or turning. Considering that balance ability is more needed when changing posture, PD patients with postural instability tend to fall during sitting/standing or turning. On the other hand, FOG is common when patients try to start walking or turn, so PD patients with FOG tend to fall forward during walking or turning.

In our study, higher NPI score was predictor for non-forward falls in PD patients. Even though various neuropsychiatric symptoms were reported to be related with falls in previous studies^8, 9^, no studies focused on the relationship between neuropsychiatric symptoms and falling subtype. Additionally, most of enrolled subjects in our study were PD patients with akinetic-rigid subtype (87.1%). Considering previous studies already suggested non-tremor dominant subtype, such as akinetic-rigid subtype or postural instability and gait disturbance subtype, to be a risk factor for falls in PD patients^11, 12^, our results were concomitant with previous studies. Even though non-tremor dominant subtype was also associated with FOG as well as postural instability^33^, akinetic-rigid subtype was predictor for falling into non-forward directions, not falling forward, in our study. Non-tremor dominant subtype is related with more severe and progressive PD^34^, and both FOG and postural instability are common in advanced PD patients. Therefore, relationship among these variables should be interpreted cautiously.

Frontal lobe dysfunction has also been associated with falls in PD patients^35^. In our results, we did not find a difference in FAB score between our two groups, and FAB score was not associated with falls in the logistic regression analysis. Considering that we did not enroll non-fallers or compare frontal lobe function between fallers and non-fallers, frontal lobe dysfunction might be the common pathophysiology for falls in PD patients, regardless of falling direction. However, frontal lobe dysfunction is a common symptom, especially in advanced PD patients, and a previous study failed to find a correlation between frontal lobe dysfunction and risk of falls in PD patients^36^. Therefore, more studies are needed to elucidate the role of frontal lobe dysfunction in PD fallers.

In this study, we enrolled recurrent PD fallers. Different clinical features have been reported between single fallers and recurrent fallers^37, 38^. Recurrent PD fallers had more severe parkinsonism^37^, and fell during different situations compared to single fallers^32^. Therefore, there may be different mechanisms involved between single and recurrent PD fallers, and PD itself might be more relevant to falling in recurrent PD fallers than in single fallers. In this study, to investigate the responsible mechanism for falls related PD itself, we enrolled PD fallers with relatively homogenous mechanism by recruiting only recurrent fallers. Additionally, because recurrent falls in PD patients are closely associated with impaired quality of life^39^, we focused on recurrent falls in PD patients in this study.

This study had some limitations. First, we did not recruit PD patients without falls or normal controls. Considering that age is a major risk factor for falls^1, 38^, it is important to rule out possible confounding effects due to aging. However, we investigated falling mechanisms in PD patients rather than in the elderly population and enrolled recurrent PD fallers in order to focus on falls associated with PD itself. Another limitation is that we dichotomized recurrent PD fallers based on their main falling direction. Considering that advanced PD patients could have both FOG and balance impairments^40^, these subjects might fall in multiple directions depending on the circumstance. In addition, the underlying mechanism for falls in PD patients could change with disease progression, but we did not check for changes in falling direction in our subjects. However, even in PD fallers with both FOG and balance impairment, there may be major pathophysiology responsible for falling. With a simple grouping strategy based on patients’ main falling direction, it might be possible to elucidate the mechanisms related to falls in each direction subgroup. In addition, several potential relevant factors associated with falls, such as orthostatic hypotension, cognitive impairment, and depression, were excluded in our study which allowed us to recruit only relatively homogenous PD fallers. Additionally, there might be possible recall bias, because we did not assess falls prospectively. To minimize the possible recall bias, we routinely checked falls (falling frequency, severity, direction and situation for falls) every 2–3 months at the Movement Disorders Clinic at Samsung Medical Center, where we enrolled all the subjects.

In conclusion, we demonstrated that FOG and balance impairment are two major mechanisms of recurrent falls in PD patients and suggest falling direction as an important and convenient predictor to identify the falling mechanism. Based on these results, a prevention strategy for falls in PD patients could be easily established based on their main falling direction.

## References

[1] Voss TS (2012). Fall frequency and risk assessment in early Parkinson’s disease. Parkinsonism Relat Disord.

[2] Bloem BR, Grimbergen YA, Cramer M, Willemsen M, Zwinderman AH (2001). Prospective assessment of falls in Parkinson’s disease. J Neurol.

[3] Lindholm B, Hagell P, Hansson O, Nilsson MH (2015). Prediction of falls and/or near falls in people with mild Parkinson’s disease. PLoS One.

[4] Wood BH, Bilclough JA, Bowron A, Walker RW (2002). Incidence and prediction of falls in Parkinson’s disease: a prospective multidisciplinary study. J Neurol Neurosurg Psychiatry.

[5] Bloem, B. R., Munneke, M., Carpenter, M. G. & Allum, J. H. The impact of comorbid disease and injuries on resource use and expenditures in parkinsonism. *Neurology* **61**, 1023; author reply 1023–1024 (2003). 10.1212/01.wnl.0000082160.30833.50 14557594

[6] Gazibara T (2015). Health-related quality of life in patients with Parkinson’s disease: Implications for falling. Parkinsonism Relat Disord.

[7] Rudzinska M (2013). Causes and consequences of falls in Parkinson disease patients in a prospective study. Neurol Neurochir Pol.

[8] Bryant MS (2012). The relation of falls to fatigue, depression and daytime sleepiness in Parkinson’s disease. Eur Neurol.

[9] Pasman EP, Murnaghan CD, Bloem BR, Carpenter MG (2011). Balance problems with Parkinson’s disease: are they anxiety-dependent?. Neuroscience.

[10] Wielinski CL, Erickson-Davis C, Wichmann R, Walde-Douglas M, Parashos SA (2005). Falls and injuries resulting from falls among patients with Parkinson’s disease and other parkinsonian syndromes. Mov Disord.

[11] Hiorth YH, Lode K, Larsen JP (2013). Frequencies of falls and associated features at different stages of Parkinson’s disease. Eur J Neurol.

[12] Paul, S. S. *et al*. Two-Year Trajectory of Fall Risk in People With Parkinson Disease: A Latent Class Analysis. *Arch Phys Med Rehabil* **97**, 372-379 e371 (2016). 10.1016/j.apmr.2015.10.105 PMC4769916 26606871

[13] Nevitt MC, Cummings SR (1993). Type of fall and risk of hip and wrist fractures: the study of osteoporotic fractures. The Study of Osteoporotic Fractures Research Group. J Am Geriatr Soc.

[14] Jankovic J (1990). Variable expression of Parkinson’s disease: a base-line analysis of the DATATOP cohort. The Parkinson Study Group. Neurology.

[15] Freeman R (2011). Consensus statement on the definition of orthostatic hypotension, neurally mediated syncope and the postural tachycardia syndrome. Clin Auton Res.

[16] Hoehn MM, Yahr MD (1998). Parkinsonism: onset, progression, and mortality. Neurology.

[17] Kang GA (2005). Clinical characteristics in early Parkinson’s disease in a central California population-based study. Mov Disord.

[18] Tomlinson CL (2010). Systematic review of levodopa dose equivalency reporting in Parkinson’s disease. Mov Disord.

[19] Giladi N (2000). Construction of freezing of gait questionnaire for patients with Parkinsonism. Parkinsonism Relat Disord.

[20] Tinetti ME, Williams TF, Mayewski R (1986). Fall risk index for elderly patients based on number of chronic disabilities. Am J Med.

[21] Dubois B, Slachevsky A, Litvan I, Pillon B (2000). The FAB A frontal assessment battery at bedside. Neurology.

[22] Cummings JL (1994). The Neuropsychiatric Inventory comprehensive assessment of psychopathology in dementia. Neurology.

[23] Kerr GK (2010). Predictors of future falls in Parkinson disease. Neurology.

[24] Matinolli M, Korpelainen JT, Sotaniemi KA, Myllyla VV, Korpelainen R (2011). Recurrent falls and mortality in Parkinson’s disease: a prospective two-year follow-up study. Acta Neurol Scand.

[25] Bloem BR, Hausdorff JM, Visser JE, Giladi N (2004). Falls and freezing of gait in Parkinson’s disease: a review of two interconnected, episodic phenomena. Mov Disord.

[26] Okuma Y (2014). Freezing of gait and falls in Parkinson’s disease. J Parkinsons Dis.

[27] Schlenstedt C (2016). Postural control and freezing of gait in Parkinson’s disease. Parkinsonism Relat Disord.

[28] Christofoletti G, McNeely ME, Campbell MC, Duncan RP, Earhart GM (2016). Investigation of factors impacting mobility and gait in Parkinson disease. Hum Mov Sci.

[29] Weaver TB, Robinovitch SN, Laing AC, Yang Y (2016). Falls and Parkinson’s Disease: Evidence from Video Recordings of Actual Fall Events. J Am Geriatr Soc.

[30] Schlenstedt C (2016). Comparison of the Fullerton Advanced Balance Scale, Mini-BESTest, and Berg Balance Scale to Predict Falls in Parkinson Disease. Phys Ther.

[31] Sparrow D (2016). Highly Challenging Balance Program Reduces Fall Rate in Parkinson Disease. J Neurol Phys Ther.

[32] Shen X, Mak MK (2015). Technology-assisted balance and gait training reduces falls in patients with Parkinson’s disease: a randomized controlled trial with 12-month follow-up. Neurorehabil Neural Repair.

[33] Forsaa EB, Larsen JP, Wentzel-Larsen T, Alves G (2015). A 12-year population-based study of freezing of gait in Parkinson’s disease. Parkinsonism Relat Disord.

[34] van der Heeden JF (2016). Postural instability and gait are associated with severity and prognosis of Parkinson disease. Neurology.

[35] Kataoka H, Tanaka N, Saeki K, Kiriyama T, Ueno S (2014). Low frontal assessment battery score as a risk factor for falling in patients with Hoehn-Yahr Stage III Parkinson’s disease: a 2-year prospective study. Eur Neurol.

[36] Lord, S. *et al*. Predicting first fall in newly diagnosed Parkinson’s disease: Insights from a fall-naive cohort. *Mov Disord* (2016). 10.1002/mds.26742 PMC5880273 27621153

[37] Mak MK, Pang MY (2010). Parkinsonian single fallers versus recurrent fallers: different fall characteristics and clinical features. J Neurol.

[38] Deandrea S (2010). Risk factors for falls in community-dwelling older people: a systematic review and meta-analysis. Epidemiology.

[39] Gazibara, T. *et al*. Health-related quality of life as a predictor of recurrent falling in Parkinson’s disease: 1-year follow-up study. *Psychogeriatrics* (2016). 10.1111/psyg.12178 26756787

[40] Pelykh O, Klein AM, Botzel K, Kosutzka Z, Ilmberger J (2015). Dynamics of postural control in Parkinson patients with and without symptoms of freezing of gait. Gait Posture.

